# ^18^F- FDG PET/CT helps differentiate autoimmune pancreatitis from pancreatic cancer

**DOI:** 10.1186/s12885-017-3665-y

**Published:** 2017-10-23

**Authors:** Jian Zhang, Guorong Jia, Changjing Zuo, Ningyang Jia, Hui Wang

**Affiliations:** 1 Department of Nuclear Medicine, Xinhua Hospital Affiliated to Shanghai Jiaotong University School of Medicine, Shanghai, 200092 China; 2 Department of Nuclear Medicine, Changhai Hospital, Second Military Medical University, Shanghai, 200433 China; 3 Department of Radiology, Eastern Hepatobiliary Surgery Hospital, Second Military Medical University, Shanghai, 200433 China

**Keywords:** Autoimmune pancreatitis, Pancreatic cancer, Positron-emission tomography

## Abstract

**Background:**

^18^F-FDG PET/CT could satisfactorily show pancreatic and extra-pancreatic lesions in AIP, which can be mistaken for pancreatic cancer (PC). This study aimed to identify ^18^F-FDG PET/CT findings that might differentiate AIP from PC.

**Methods:**

FDG-PET/CT findings of 26 AIP and 40 PC patients were reviewed. Pancreatic and extra-pancreatic lesions related findings, including maximum standardized uptake values (SUVmax) and patterns of FDG uptake, were identified and compared.

**Results:**

All 26 patients with AIP had increased pancreatic FDG uptake. Focal abnormal pancreatic FDG activities were found in 38/40 (95.00%) PC patients, while longitudinal were found in 18/26 (69.23%) AIP patients. SUVmax was significantly different between AIP and PC, both in early and delayed PET/CT scans (*p* < 0.05). AUCs were 0.700 (early SUVmax), 0.687 (delayed SUVmax), 0.683 (early lesions/liver SUVmax), and 0.715 (delayed lesion/liver SUVmax). Bile duct related abnormalities were found in 12/26 (46.15%) AIP and 10/40 (25.00%) PC patients, respectively. Incidentally, salivary and prostate gland SUVmax in AIP patients were higher compared with those of PC patients (*p* < 0.05). In males,an inverted “V” shaped high FDG uptake in the prostate was more frequent in AIP than PC patients (56.00%, 14/25 vs. 5.71%, 2/35). Increased FDG activity in extra-pancreatic bile duct was present in 4/26 of AIP patients, while was observed in none of the PC patients. Only in AIP patients, both diffuse pancreatic FDG accumulation and increased inverted “V” shaped FDG uptake in the prostate could be found simultaneously.

**Conclusions:**

^18^F-FDG PET/CT findings might help differentiate AIP from PC.

## Background

Autoimmune pancreatitis (AIP) is a chronic pancreatitis characterized by pancreatic enlargement, irregular pancreatic duct stenosis, and increased serum IgG4 levels, mediated by autoimmune mechanisms [1]. Although AIP responds well to steroid therapy, it has no characteristic clinical manifestation(s) and may easily be misdiagnosed as pancreatic cancer (PC) or cholangiocarcinoma, with patients having to undergo unnecessary surgeries and sustaining hardship and high expenses [2, 3]. About 2.2% of lesions resected with suspected pancreatic carcinoma are histologically proven AIP [4]. Vice versa, 95.7% (22/23) of AIP patients are misdiagnosed with pancreatic cancer or bile duct cancer [5], with up to 91.3% (21/23) operated. FDG PET/CT imaging, with high sensitivity, can show characteristic glucose metabolism that reflects the inflammatory activity of pancreatic lesions [6]. It has been reported that FDG PET/CT could satisfactorily show pancreatic and extra-pancreatic lesions in AIP patients, providing more specific information for diagnosis and facilitating the understanding of AIP’s pathological features [7–11]. Besides, in previous studies [12, 13], we found that more than half of AIP patients show inverted “V” shaped high FDG uptake in the prostate. Further research is needed to determine whether the metabolic characteristics of the pancreas and extra-pancreatic organs could be used for the differential diagnosis of AIP and PC.

## Methods

### Patient population

In this retrospective study, the patients in our study were **s**elected from the population who were suspected of having pancreatic mass and underwent FDG PET/CT. Consecutive patients diagnosed with AIP from August 2010 to March 2014 at Changhai hospital were analyzed. The PC group comprised randomly selected age and sex matched patients to match the AIP group.

Inclusion criteria were: (1) confirmed diagnosis of AIP based on the 14th International Association of Pancreatology diagnostic criteria (which include 5 aspects, namely pancreatic and main pancreatic duct images, serology, EPLs, histology, and hormone therapeutic reaction) and ^18^F-FDG PET/CT imaging results before treatment for AIP; (2) confirmed diagnosis of pancreatic cancer based on histological findings or liquid based cytology.

Exclusion criteria were: (1) invasive examinations such as aspiration biopsy, ERCP, and stent placement before the PET/CT examination; (2) treatment for inflammation or cancer.

Of the 36 enrolled AIP patients, 10 were excluded (5, 2, and 3 had a history of acute pancreatitis within prior 6 months, incomplete PET/CT, and a recent history of ERCP and/or biliary stent placement, respectively). Median age of the 26 AIP patients included was 60 years, ranging from 40 to 83 years; there was one female. Meanwhile, a total of 40 patients with pancreatic cancer (35 men and 5 women, aged 34-82 years, median age of 60 years) were enrolled. The study was approved by the ethics committee of Chnaghai hospital.

Each of the 66 patients (26 AIP and 40 PC) underwent a whole body PET/CT. Meanwhile, 22/26 AIP patients and 36/40 PC patients had additional delayed PET-CT scans of the abdomen at 120 min after tracer injection.

### PET/CT scan

The Siemens Biograph64 PET/CT (52 LSO crystal and 64-slice spiral CT) was used for the PET/CT. ^18^F-FDG (radiochemical purity >95%) was provided by Shanghai Atomic Sinovac Pharmaceutical Co., Ltd. Subjects were instructed to fast for more than 6 h, and 3.70-5.55 MBq/kg of ^18^F-FDG was intravenously injected when blood glucose (BG) < 11.1 mmol/L. Then, after resting in the waiting room for 60 min, a body topogram scan was performed using an electric current of 35 mA at a voltage of 120 kV, a scan time of 10.5-15.6 s and a scan thickness of 0.6 mm. Then, whole-body CT scans were performed using an electric current of 170 mA at a voltage of 120 kV, with a scan time of 18.67-21.93 s and scan thickness of 3 mm. Then, whole-body PET scans were performed covering 5-6 bed positions, with an acquisition time of 2.0-2.5 min per bed position. The 3D scanning of the head was performed additionally. Delayed PET scans of the pancreas were carried out with 1-2 bed positions,120 min after injection with ^18^F-FDG, using the same parameters described above. Images were reconstructed by the post-processing workstation TureD System, including the direction of cross-sectional, coronal, and sagittal tomographic images and three-dimensional projection images.

### Image analysis

^18^F-FDG PET/CT images were interpreted by two experienced nuclear medicine physicians blinded to clinical and histopathological data. Images were evaluated by visual, subjective and semi-quantitative (SUVmax) methods. Any disagreement was resolved by discussions. Mean values from both physicians were considered as final results. The mean retention index (RI) was calculated as RI = ([PET_120min_ SUVmax] – [PET _60min_ SUVmax] ÷ PET _60min_ SUVmax ×100%.

The SUVmax values of lesions of the pancreas, hilar lymph nodes, peri-pancreatic lymph nodes, liver, salivary gland, and prostate were measured. SUVmax ratio of prostate to liver background ratio (PBR) was calculated. Pancreatic lesions were grouped into three categories: a) diffuse, b) focal, and c) multifocal. The multifocal type was defined as more than 1 foci of non-continuous pancreatic lesions were present. The presence or absence of each of the following parameters was determined as “yes” (present) or “no” (absent) by the reviewers: (1) increased FDG activity in the pancreatic lesion; (2) dilated main pancreatic duct; (3) biliary duct abnormalities, including increased FDG activity in the extra-pancreatic bile duct and gallbladder, dilation and wall thickening of intra and extra-hepatic bile ducts; (4) abnormal mediastinal, pulmonary hilar, peri-pancreatic or retroperitoneal lymph nodes; (5) retroperitoneal fibrosis; (6) inverted “V” shaped high FDG uptake in the prostate.

### Statistical analysis

Statistical analyses were performed with SPSS version 17.0. Measurement data with normal distribution were shown as $\overline{x}$ ± SD, and compared by independent samples *t*-test. Data with abnormal distribution were presented in median and interquartile range (IQR), and compared by rank-sum test. Using statistically significant data of the above parameters, ROC curves were generated and areas under the curves (AUCs) were calculated. Cut-off values were determined by the Youden index. The corresponding sensitivity, specificity, and positive predictive value were calculated. Differences in SUVmax between PET_60min_ and PET_120min_ scans were analyzed by *t*-test, and the corresponding SUVmax retention index calculated. Differences in detection rates between the two groups were determined using chi-squared analysis or Fisher’s exact test.

## Results

The 26 AIP patients included 25 men and 1 woman with a median age of 60.0 ± 10.7; two patients were diagnosed based on IgG4-positive plasma cells found by submandibular lymph node and pancreatic biopsies, respectively. In 17 patients, endoscopic ultrasonography assisted fine-needle aspiration revealed no malignant cells. Meanwhile, 22 patients had abnormally elevated serum IgG4 levels. All 26 patients had clinical follow-up with imaging studies at least 6 months.

The forty pancreatic cancer patients included 35 men and 5 women with a median age of 60.7 ± 10.6; final diagnosis was confirmed by surgical pathology (*n* = 23) and endoscopic ultrasonography assisted fine-needle aspiration (FNA) and/or laparoscopy (*n* = 17). In PC group, diameter of the tumour was 30.9 ± 12.2 mm, and 57.5% (23/40) located in the pancreatic head, 22.5% (9/40) located in the pancreatic body, 10% (4/40) located in the pancreatic tail, 10% (4/40) located in the junction of pancreatic body and tail; 20% (8/40) was classified as Stage T1, 10% (4/40) was classified as Stage T2, 30% (12/40) was classified as Stage T3, 40% (4/40) was classified as Stage T4; 50% (20/40) was classified as Stage N0, 50% (20/40) was classified as Stage N1; 72.5% (29/40) was classified as Stage M0, 27.5% (11/40) was classified as Stage M1.

Clinical characteristics of AIP and PC groups were shown in Table 1. There was no statistical difference between the two groups, except for CA19-9 and ALP.

**Table 1:** Clinical characteristics of patients with autoimmune pancreatitis and pancreatic cancer

	AIP group *n* = 26	PC group *n* = 40	*P* value
Women / men	1/25	5/35	NS
Age (years)	60.03 ± 10.72	60.73 ± 10. 60	NS
Fasting blood sugar (mmol/L)	5.90 ± 1.40	6.10 ± 1.47	NS
CRP (mg/L)	5.66(3.32, 8.69)	11.15 ± 7.44	NS
White blood cells (×10^9^/L)	6.15 ± 1.37	5.65 ± 2.12	NS
BUN (mmol/L)	5.24 ± 2.51	5.26 ± 1.80	NS
Creatinine (μmol/L)	70.00(57.25, 79.25)	72.87 ± 16.01	NS
Total bilirubin (μmol/L)	11.60(7.00, 23.90)	14.10(10.60, 21.60)	NS
ALP (U/L)	214.95 ± 164.52	84.00(61.00, 118.00)	*P* < 0.05
Amylase (U/L)	130.63 ± 157.74	49.00(39.75, 135.00)	NS
CA19-9 (U/ml)	18.30(7.72, 71.63)	406.81 ± 352.09	*P* < 0.05
Serum total protein(g/L)	62.88 ± 19.99	68.69 ± 5.72	NS
Serum albumin(g/L)	33.59 ± 6.63	39.54 ± 3.87	*P* < 0.05
Serum globulin(g/L)	33.41 ± 8.97	29.15 ± 4.08	NS
Albumin/Globulin	1.05 ± 0.28	1.38 ± 0.20	*P* < 0.05
ALT(U/L)	80.71 ± 104.02	23.00(14.00,39.00)	NS
AST(U/L)	62.23 ± 60.98	23.00(19.00,27.00)	*P* < 0.05

*CRP* C-reactive protein, *BUN* blood urea nitrogen, *ALP* alkaline phosphatase, *ALT* Alanine aminotransferase, *AST* Aspartate aminotransferase

### Findings of whole body ^18^F-FDG PET/CT studies

#### Pancreatic lesions (Tables 2, 3 and 4)

All patients had increased FDG activity of pancreatic lesions on early PET/CT scan (PET_60min_). Eighteen patients with a diffuse pattern of increased pancreatic FDG uptake, six patients with a focal FDG uptake lesion and two patients with multifocal pattern were observed in 26 AIP patients. Average SUVmax was 5.24 ± 1.81.

**Table 2:** Comparison of quantitative metabolic parameters between the AIP and PC groups

Groups	AIP group	PC group	*P* value
Early SUVmax of pancreatic lesions	5.24 ± 1.81	7.30 ± 3.21	0.001*
Early SUVmax of Liver	2.84 ± 0.50	2.90 ± 0.40	0.64
Pancreas lesion/liver in early scan	1.91 ± 0.83	2.57 ± 1.17	0.015*
Delayed SUVmax of pancreatic lesions	6.54 ± 2.41	9.15 ± 4.89	0.004*
Delayed SUVmax of Liver	2.73 ± 0.52	2.61 ± 0.53	0.407
Pancreas lesion/liver in delayed scan	2.48 ± 1.10	3.48 ± 1.49	0.005*
RI of Pancreas lesion	21.32 ± 13.11	21.23 ± 24.94	0.986
RI of liver	−1.81 ± 6.46	−6.71(−9.90,-1.18)	0.047*
SUVmax of salivary gland	2.36(1.95,3.41)	2.02 ± 0.76	0.003*
Mediastinal/hilar lymph node	3.52(2.46,4.67)	2.77(2.48,3.99)	0.198
Peri-pancreatic lymph node	2.03 ± 1.23	2.28(1.31,3.78)	0.27
SUVmax of prostate	3.11 ± 1.27	2.11 ± 0.44	0.01*
Prostater/liver	1.10 ± 0.45	0.73 ± 0.15	0.001*

*P < 0.05

**Table 3:** Performance of multiple metabolic parameters in differential diagnosis of AIP and PC

Diagnostic parameters	AUC	Cutoff value	Sensitivity	Specificity	Accuracy
Early SUVmax of pancreatic lesions	0.700	5.94	70.0%	76.9%	72.7%
Pancreas lesion/liver in early scan	0.683	2.16	70.0%	73.1%	71.2%
Delayed SUVmax of pancreatic lesions	0.687	8.13	63.9%	86.4%	72.4%
Pancreas lesion/liver in delayed scan	0.715	3.14	69.4%	90.9%	77.6%
SUVmax of salivary gland	0.716	1.92	84.6%	57.5%	68.2%
SUVmax of prostate	0.776	2.94	56.0%	97.1%	80.0%
RI of liver	0.657	−5.87%	72.7%	58.3%	63.8%
Prostater/liver	0.729	1.02	56.0%	97.1%	80.0%

**Table 4:** Common PET-CT findings in patients with autoimmune pancreatitis and pancreatic cancer

PET/CT findings	AIP group	PC group	*P* value
Dilated pancreatic duct	6/26	22/40	*P* < 0.05
Changes of biliary system	12/26	10/40	*P* < 0.05
High uptake of extra-pancreatic bile duct	4/26	0/40	*P* < 0.05
Mediastinal & hilar lymph node	17/26	20/40	NS
Peri-pancreatic and peritoneal lymph node	20/26	31/40	NS
Inverted “V” shape high prostate FDG uptake	14/25	2/35	*P* < 0.001
Retroperitoneal fibrosis	2	0	NS

In the PC group (*n* = 40), the patterns of increased FDG metabolic activity included: focal (*n* = 38), diffuse (*n* = 1), multifocal (*n* = 1) types, with an average SUVmax of 7.30 ± 3.21. FDG activity of the lesions in PC patients was higher than that of the AIP group, with a statistically significant difference (*t* = −3.32, *P* < 0.05).

Twenty-two patients with AIP and 36 patients with PC had delayed PET/CT scans (PET_120min_), with a statistically significant difference (*t* = −2.967, *P* < 0.05) between average SUVmax between the two groups.

We also compared liver SUVmax and the ratio of pancreatic lesion SUVmax to liver SUVmax between the two groups in both early and delayed scans. The results showed that SUVmax of pancreatic lesion and above SUVmax ratio were statistically different. However, SUVmax of the liver were not significantly different between AIP and PC patients (Table 1).

ROC curves showed that the AUC of the ratio of pancreatic lesion SUVmax to liver SUVmax in delayed scan was the largest (0.715) to diagnose PC.

AIP patients’ pancreatic lesion SUVmax RI was 21.32 ± 13.11%, while 21.23 ± 24.94% was obtained for PC patients. Liver SUVmax RI values were −1.81 ± 6.46 and −6.71% (−9.90, −1.18%) in AIP and PC patients, respectively. There was statistically significant difference between the two groups in the RI values of the liver (Mann-Whitney U test, *P* < 0.05), but not of pancreatic lesions (*P* > 0.05) (Table 1).

Pancreatic duct dilatation was observed in only 6 of 26 (23.1%) AIP patients, and in 22 of the 40 (55.0%) patients with PC. Fisher’s exact test confirmed the statistically significant difference between the two groups (*P* < 0.05).

### Extra-pancreatic lesions (EPLs) on PET/CT

#### Morphological and metabolic features of EPLs (Table 3)

**Fig. 1:**
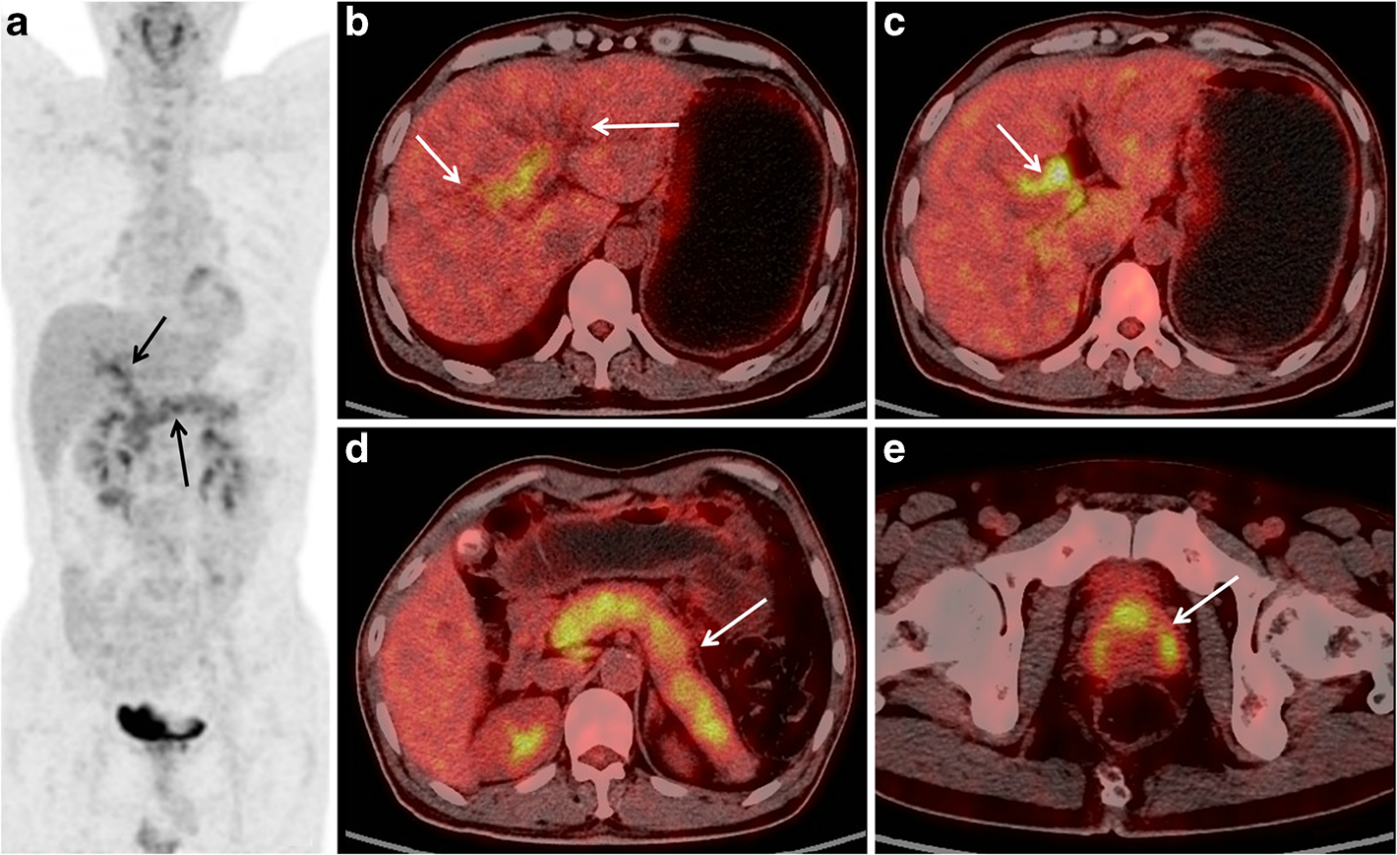
A 55-years old male patient with AIP. The MIP PET image (**a**) shows a diffuse and heterogeneous increase of FDG uptake in the pancreas, as well as increased FDG activity along the bile duct; PET/CT fusion images (**b**) depicts bile duct dilatation; (**c**), increased FDG activity of the hilar bile duct is shown; (**d**) shows diffusely enlarged pancreas with capsule-liked rim and a heterogeneous increase of FDG uptake. **e**, there is an inverted “V” shaped high FDG uptake in the prostate

**Fig. 2:**
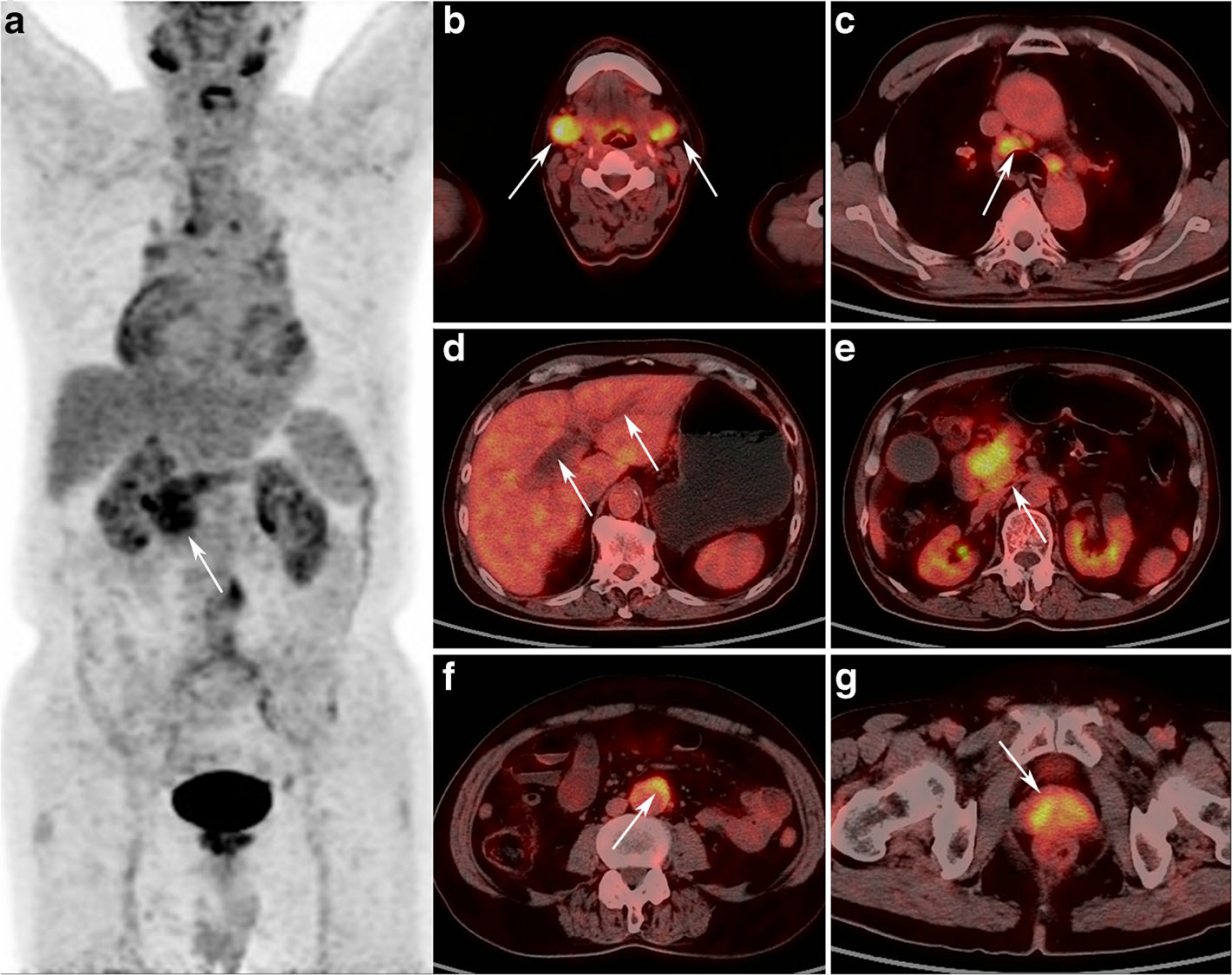
A 66-years old male patient with focal AIP in the pancreatic head. MIP PET (**a**) and PET/CT fusion (**e**) images shows localized enlargement of the pancreatic head with increased FDG uptake (arrow), with early and delayed SUVmax of 6.7 and 8.0, respectively. PET/CT fusion images shows (**b**) increased FDG uptake in bilateral submandibular gland, with a SUVmax of 7.9; (**c**), enlargement of mediastinal lymph node with increased FDG uptake (SUVmax, 5.7); (**d**), dilatation of bile duct; (**f**), retroperitoneal fibrosis around artery; (**g**) inverted “V” shaped high FDG uptake in the prostate

**Fig. 3:**
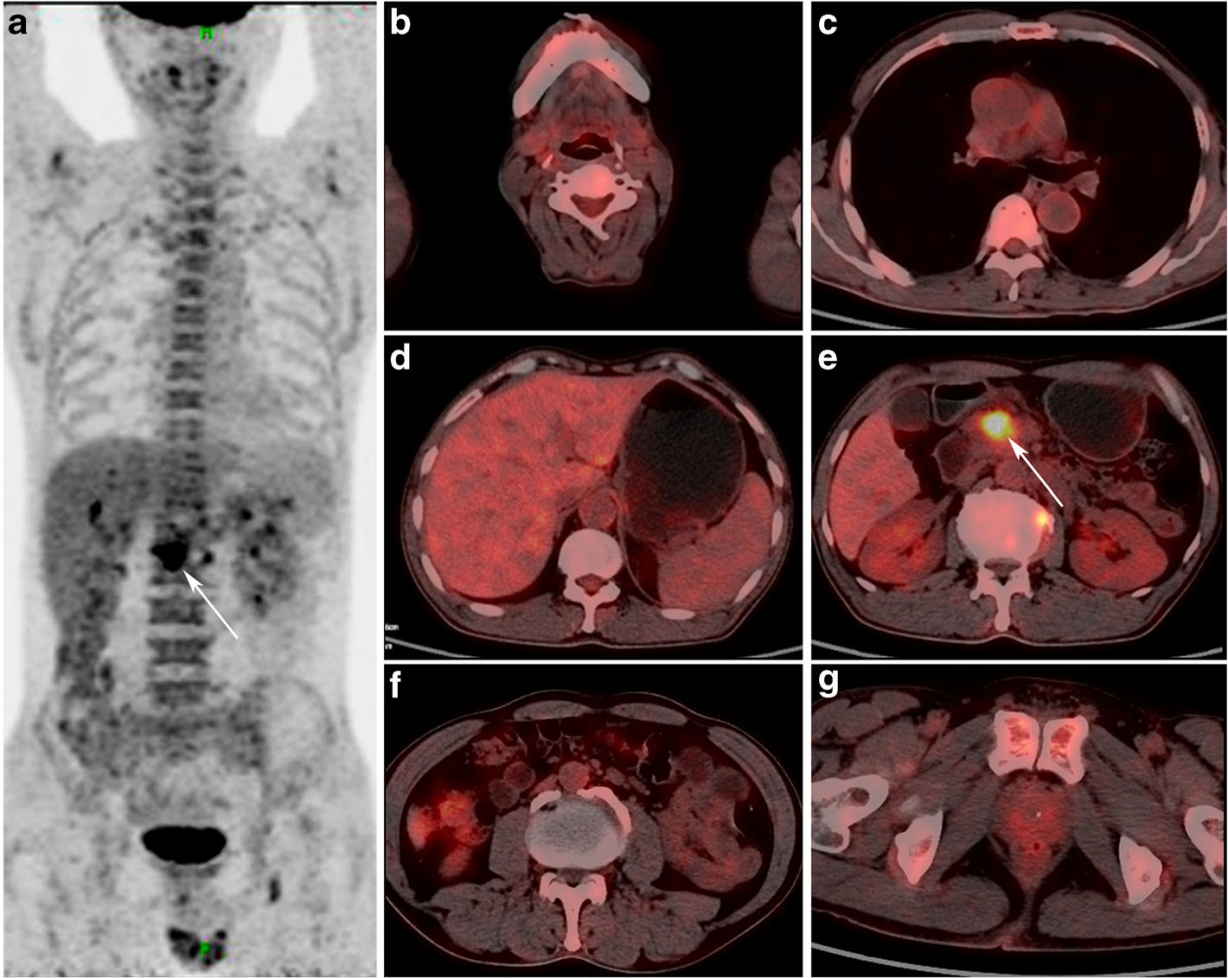
A 59-years old male patient with pancreatic cancer. MIP PET (**a**) and PET/CT fusion (**e**) images show a mass in the pancreatic head with increased FDG uptake (arrow), with early and delayed SUVmax of 9.3 and 10.8, respectively. Compared with AIP patients in Fig. 2, no increased FDG uptake foci in the salivary gland **b**), mediastinal lymph nodes (**c**), retroperitoneal space (**f**), and prostate (**g**) are observed, as well as no bile duct expansion (**d**)

Fisher’s exact test showed statistical differences in the positive rates of high FDG uptake of extra-pancreatic bile duct (*P* < 0.05) and inverted “V” shaped high FDG uptake in the prostate (*P* < 0.001); however, there was no statistically significant difference in the positive rate of retroperitoneal fibrosis, FDG uptake of mediastinal and hilar lymph node, or peri-pancreatic peritoneum lymph nodes. Increased FDG activity of the extra pancreatic portion of the bile duct was only observed in the AIP group (4/26 of patients), but not in PC patients (0/40); with pancreatic lesions showing diffuse FDG accumulation, inverted “V” shaped high FDG uptake in the prostate was observed in 11 of the 20 patients with AIP, while none of the 2 patients with PC showed this feature (Figs. 1, 2 and 3).

### Quantitative analysis of PET/CT in extra-pancreatic lesions (Tables 1, 2)

#### Salivary glands

Median SUVmax of salivary glands in AIP patients was 2.36 (range: 1.95- 3.41), and was 2.02 ± 0.76 in PC patients, indicating a statistically significant difference between the two groups (*P* < 0.05). The AUC of the SUVmax of salivary glands in diagnosing AIP was 0.716; with a cut-off value of 1.92, sensitivity and specificity were 84.6 and 57.5%, respectively.

### Lymph nodes

Median SUVmax of mediastinal and hilar lymph nodes in AIP patients was 3.52 (range: 2.46-4.67) and was 2.77 (range: 2.48-3.99) in PC patients. No significant difference between the two groups was found (Mann-Whitney U test; *P* > 0.05). No significant difference in the average SUVmax of peri-pancreatic and retroperitoneum lymph nodes was observed between the two groups (2.28 (1.31-3.78) for patients with PC, 2.03 ± 1.23 for patients with AIP, *P* > 0.05).

### Prostate

Mean SUVmax of the prostate was higher in the AIP group (3.11 ± 1.27) than that in the PC group (2.11 ± 0.44, *P* < 0.05). The AUC of prostate SUVmax in diagnosing AIP was 0.776; with a cut-off value of 2.94; sensitivity, specificity, and accuracy value were 56.0, 97.1, and 97.1%, respectively.

The ratios of prostate SUVmax to liver SUVmax (prostate/liver) were also evaluated. Compared with the PC group (0.73 ± 0.15), AIP patients showed significantly higher values (1.10 ± 0.45; *t* = −4.584, *P* < 0.05). AUC of this ratio in diagnosing AIP was 0.729; with a cut-off value of 1.02, sensitivity, specificity, and accuracy value were 56.0, 97.1, and 97.1%, respectively.

## Discussion

Autoimmune pancreatitis (AIP) is a specific type of chronic pancreatitis, including two subtypes, which might be identified using tissue pathology, clinical features and/or diagnostic criteria [14–16]. Type-I AIP is more prevalent in elderly Asian males and characterized by lymphoplasmacytic sclerosing pancreatitis, which commonly involves other organs as well.

In the early years, due to the lack of awareness of AIP, it was often misdiagnosed as PC. In a study of 37 AIP patients utilizing conventional imaging modalities, six patients were misdiagnosed as PC and two cases as cholangiocarcinoma [17]. Another study found that 9 of 17 AIP cases were misdiagnosed as pancreatic cancer, and they proposed a few reasons: demographics, clinical manifestations, serology and bile duct stenosis [18]. With the wide application and development in recent years, contrast-enhanced CT and MR have played an important role in the differential diagnosis of AIP and PC. Some manifestation, such as “sausage-like” pancreatic enlargement, capsule-like rim, segmental stricture of pancreatic duct, and delayed enhancement were known as the characteristics of AIP. In this study, we found some parameters and imaging characteristics of FDG PET/CT could help to differentially diagnose AIP from PC.

It is well documented that increased FDG accumulation might be used as a marker of inflammatory lesions [19–21]. Thus, FDG PET/CT might play a critical role in revealing pancreatic and extra-pancreatic lesions in patients with AIP. Ozaki et al., [9] showed that high FDG activity was observed in all AIP patients, while only 73.1% PC patients had increased FDG activity in lesions.

A focal, nodular pattern of increased FDG activity was significantly more frequent in patients with pancreatic cancer, whereas an longitudinal abnormal pancreatic FDG activities was more suggestive of AIP [8, 9]. In this study a longitudinal pattern of increased FDG accumulation along the pancreas was found in 69.2% of AIP patients, and only in 2.5% of PC cases. On the other hand, a focal nodular pattern of increased FDG activity was found in 23.1 and 95% patients with AIP and PC, respectively.

Previous studies suggested that FDG SUV of a lesion was usually greater than 4.0 in patients with PC, 3.0 - 4.0 in chronic pancreatitis patients and below 3 in healthy volunteers [22]. Although AIP is a subtype of chronic pancreatitis, Ozaki et al. [9] found no significant difference in SUV of lesions between patients with AIP and PC, either on early or delayed phase images; similar SUVmax ratio between early and delayed phases were also found. Lee et al. [8] compared the frequency of increased FDG uptake of pancreatic lesions in patients with AIP and PC, and overall frequency showed no significant differences as well. Our study, including age and sex matched AIP (*n* = 26) and PC (*n* = 40) patients, suggested that both SUVmax and the SUVmax ratio of pancreas to liver in the early and delayed phases were significantly different. On early PET/CT scans with a cut-off value of SUVmax of 5.94, diagnostic sensitivity and specificity were 70.0 and 76.9%, respectively, for PC diagnosis. The larger sample size of AIP in the present study might be the reason for disagreement with prior reports. In addition, the following reasons could be considered as well. (1) Technical differences between previously used PET scanners and modern PET-CT scanners, which could account for discrepant SUV values. (2) In the current study, FDG SUV were compared in age and sex matched two groups, unlike previous studies [8, 9]. (3) In the current study, the AIP patient population comprised consecutive patients, which was not the case in Lee et al. [8]. (4) The number of pancreatic cancer patients in Lee’s [8] study was distinctly larger than that of AIP patients, which might have impaired the power of statistical tests. (5) Invasive diagnostic procedures were performed prior to PET/CT scan in some cases, which might have influenced FDG metabolism and the SUV value. In the current study such factors were well controlled and eliminated. However, the diagnostic sensitivity of SUV in the PC group was lower (70.0%) in the current study. We tentatively put forward that diversity of PC metabolism results in varied SUV values in a large range. For example, FDG uptake may increase slightly in some small, low-malignant or mucinous pancreatic cancer cases. Furthermore, a quite remarkable metabolism was detected in some patients with AIP, with the highest SUVmax of 11.8, leading to false positivity and decreased specificity.

Patients with AIP may have autoimmune inflammation involving the liver and biliary tract [23, 24]; inflammation induces abnormal hepatobiliary function and decreases the liver clearance of FDG. Our results revealed that FDG retention index values of the AIP and PC groups were 1.8 and 6.7%, respectively, suggesting that liver clearance of FDG activity is slower in the AIP group than in PC patients. Thus, the AIP SUVmax ratio of lesion to liver was lower compared with that of the PC group in the delayed phase.

When pancreatic cancer invades the bile duct, the main manifestation is direct compression of the bile duct, stenosis, or even an obstruction. In pancreatic cancer, the bile duct can also be compressed by a metastatic lymph node. In AIP patients, the inflammatory swelling of the pancreatic head could also cause narrowing and occlusion of the distal common bile duct, eventually leading to secondary expansion/dilatation of the upstream bile duct. In the current study, 46.2% patients in the AIP group showed biliary duct changes, which was only present in 25.0% of PC patients. The higher positive rate for the AIP group is possibly related to frequent involvement of the pancreatic head (88.5 AIP vs 52.5% PC). Besides concomitant bile duct inflammation might result in higher proportion of biliary system change in AIP patients. Moreover, this study revealed that FDG accumulation in the extra pancreatic portion of the bile duct was only observed in AIP patients.

A number of studies have shown that AIP also involves salivary glands, and is characterized by increased FDG hyper metabolism on PET/CT. Ozaki’s comparative study [9] found that 13.3% (2/15) of AIP patients have FDG accumulation in the salivary gland, with the PC group showing no increased FDG accumulation in the salivary gland. Lee et al. [8] showed that 35.3% (6/17) of AIP patients have high FDG accumulation in the salivary gland, with statistically significant difference between the two groups. In this study, considering physiological FDG uptake of normal salivary gland, we measured the SUVmax of the salivary gland, and found higher values in the AIP group compared with PC patients (2.36 vs 2.02, *P* < 0.05). At a threshold value for salivary gland’s FDG SUVmax of greater than 1.92, diagnostic sensitivity was 84.6% in the AIP group, for a specificity of 57.5%. These results suggested the involvement of the salivary gland in patients with AIP.

Our previous studies demonstrated that over half of AIP male patients show inverted “V” shaped high FDG uptake in the region of the prostate gland [12, 13]. This characteristic may be related to inflammatory infiltration of the prostatic transitional and central zones, which shaped like a inverted “V”. However, whether this characteristic can be used to differentiate AIP from PC remains unclear. This study found that the positive rate of inverted “V” shaped high FDG uptake was significantly different in AIP and PC patients (56.0 vs 2.9%). This result provided more evidence that inverted “V” shaped high FDG uptake in the prostate can contribute to AIP diagnosis. The previous reports [9, 23] of complicated prostatitis of AIP patients, but the frequency is low. Reasons may include: (1) due to normal prostate also can uptake FDG physiologically, previous reports defined prostatic involvement usually based on FDG metabolism increased significantly in prostate, and symptoms associated with prostatitis. While in our research, it is based on whether there is inverted “V” shaped high FDG accumulation, wihich might with slightly increased FDG metabolism, most of these patients had not symptoms associated with prostatitis. Two PC patients were detected with inverted “V” shaped high FDG uptake. Given their advanced ages, we speculated that these patients may have autoimmune prostatitis [25]. However, only in AIP patients, both diffuse pancreatic FDG accumulation and increased inverted “V” shaped FDG uptake in the prostate could be found simultaneously. We also found that prostate SUVmax in the AIP group was higher than in PC patients; with a cut-off value of 2.94, a specificity of 97.1% was obtained. Although previous studies [8, 9] suggested that retroperitoneal fibrosis is commonly observed in AIP patients, the current study population had a very limited number of such cases, namely 2 patients.

In contrast to prior reports [8, 9], no significant differences in SUVmax and the frequency of increased FDG uptake of mediastinal and hilar lymph nodes were found between the two groups. A possible reason is that patients with pancreas cancer sometimes suffer from mediastinal and hilar lymph node metastasis; besides, elder patients could show non-tumorous FDG accumulation in mediastinal and hilar lymph nodes [26]. This study found no statistically significant difference in SUVmax and number of para-pancreatic lymph nodes. A possible reason is that metastatic lymph nodes in pancreatic cancer might have small volume and low FDG uptake; another explanation is that AIP patients could have peri-pancreatic lymph node involvement.

## Conclusions

Diffuse uptake of FDG in the pancreas might be used to differentiate AIP from PC. Furthermore, some parameters including the ratio of pancreatic lesion/liver SUV, the SUV of salivary glands, the SUV of prostate might help to differentiate, and some morphological and metabolic features of EPLs including inverted “V” shaped high FDG uptake in the prostate and increased FDG activity in extra-pancreatic bile duct have high specificity for diagnosing AIP. FDG PET/CT could provide various findings as supplements to CECT and MR, which might improve the diagnostic accuracy.

## Abbreviations

AIP: Autoimmune pancreatitis
AUCs: Areas under the curves
EPLs: Extra-pancreatic lesions
FNA: Fine-needle aspiration
IQR: Interquartile range
PBR: prostate-to-background ratio
PC: Pancreatic cancer
RI: Retention index
SUVmax: Maximum standardized uptake values
